# The Impact of a Diabetes Local Enhanced Service on Quality Outcome Framework Diabetes Outcomes

**DOI:** 10.1371/journal.pone.0083738

**Published:** 2013-12-17

**Authors:** Sopna Choudhury, Shakir Hussain, Guiqing Yao, Jill Hill, Waqar Malik, Shahrad Taheri

**Affiliations:** 1 National Institute for Health Research (NIHR) Collaborations for Leadership in Applied Health Research and Care (CLAHRC) Birmingham and Black Country Theme 8, University of Birmingham, Birmingham, United Kingdom; 2 School of Health and Population Sciences, University of Birmingham, Birmingham, United Kingdom; 3 Primary Care and population Sciences, University of Southampton, Southampton, United Kingdom; 4 Birmingham Community Healthcare NHS Trust, Birmingham, United Kingdom; 5 Diabetes Centre, Heart of England NHS Foundation Trust, Birmingham, United Kingdom; 6 Department of Medicine, Weill Cornell Medical College in New York USA and Doha, Qatar; 7 Department of Medicine, King’s College London, London, United Kingdom; Tilburg University, Netherlands

## Abstract

**Background:**

The rising challenge of diabetes requires novel service delivery approaches. In the UK, Local Enhanced Services (LES) have been commissioned for diabetes. Health professionals from general practices (GPs) who signed up to LES were given additional training (and a monetary incentive) to improve management of patients with diabetes. All practices in the PCT were invited to the LES initiative, which ensured avoiding selection bias. The aim of the study was to examine the impact of LES in terms of diabetes Quality Outcome Framework (QOF) indicators: DM23(glycaemia), DM17(lipid) and DM12(blood pressure; BP).

**Methods:**

QOF diabetes indicators were examined using data from 76 general practices for 2009–2010 in a large primary care trust area in Birmingham, UK. Data were extracted from Quality Management Analysis System. The primary outcome was a difference in achievement of QOF indicators between LES and NLES practices. A secondary outcome was the difference between LES and non-LES practices for hospital first and follow-up appointments.

**Results:**

We did not find any difference for DM12(BP) and DM17(lipid) outcomes between LES and NLES practices. However, LES practices were more likely to achieve the DM23(glycaemia) outcome (estimated odds 1.459;95% CI:1.378-1.544; P=0.0001). The probability of achieving satisfactory level of DM23(glycaemia) increased by almost 10% when GPs belonged to LES groups compared with GPs in NLES group. LES practices were less likely to refer patients to secondary care.

**Conclusion:**

Overall, LES practices performed better in the achievement of DM23(glycaemia) and also referred fewer patients to hospital, thereby meeting their objectives. This suggests that the LES approach is beneficial and needs to be further explored in order to ascertain whether the impact exerted was due to LES.

## Introduction

The prevalence of diabetes mellitus is increasing alarmingly worldwide. The World Health Organization (WHO) estimates that over 346 million people have diabetes, and that by 2030, diabetes will become the 7^th^ leading cause of death worldwide [1]. The number diagnosed with diabetes in the UK has increased by nearly 50% since general practices (GPs) first published diabetes data in 2005 from 130,000 to 2.9 million in 2011 [2]. Diabetes micro- and macro-vascular complications reduce quality of life, and increase mortality, particularly from cardiovascular disease [3-5]. Diabetes is a major challenge to healthcare services worldwide necessitating urgent preventive action, while providing and developing accessible and effective evidence-based services to reduce the burden of diabetes.

 In April 2004, the UK National Health Service (NHS) introduced the Quality and Outcomes Framework (QOF; http://www.qof.ic.nhs.uk/) as part of the General Medical Services (GMS) contract for UK general practice [6]. The aim was to improve patient care and service delivery for chronic diseases such as diabetes through financial incentives. For diabetes, there is evidence from major studies that glycemic [7-11], blood pressure [12-14], and lipid control [15,16] will reduce the risk of complications. As part of QOF, GPs have been rewarded for the percentage of patients achieving pre-set glycemic, blood pressure, and cholesterol level targets.

 Given the increasing prevalence of chronic conditions such as diabetes, further incentivization to general practice was developed through commissioning of Enhanced Services aiming to provide a greater range of local services for patients while reducing pressures on secondary care. For diabetes, enhanced services would offer the framework and resources for providing local comprehensive diabetes management within the practice for appropriate patients, to support self-management, and to prevent diabetes complications. This included the appropriate use of the community diabetes team and secondary care as per the PCT diabetes guidelines and agreed secondary care referral criteria.

The practices were required to provide, completely in primary care, the diabetes management for all patients who did not meet the referral criteria for secondary care. This included those with Type 2 diabetes controlled with lifestyle alone or oral hypoglycaemic agents and patients with Type 2 diabetes requiring insulin treatment and patients with stable Type 1 diabetes. 

In order to meet the service aims, the practices were required to review all patients currently managed in secondary care, and discharge all patients who do not meet the referral criteria who were receiving routine diabetes care from secondary care providers, and provide this care in the practice (with negotiated support from the community diabetes service). As part of this, patients were offered dedicated protected diabetes clinic time at least twice a year, of which one visit was to be for the annual diabetes needs assessment and development of the agreed diabetes care and management plan with the diabetes accredited GP. 

The impact of commissioned locally enhanced services (LES) on diabetes outcomes has not been previously investigated. We examined the LES impact on QOF diabetes outcomes in a large UK primary care trust area.

## Methods

The study used data from the Quality Management Analysis System (QMAS) with data extracted from GP patient records. We examined QOF diabetes indicators using data from 76 practices for full year of collection 2009–2010 from Birmingham East and North Primary Care Trust (BENPCT, Birmingham, UK) general practices. In order to identify whether any achievement was due to LES or a time difference, we examined available data from 34 Non-LES (NLES) practices in 2004-2005 and in 2009-2010.

### Outcome Measures

The primary aim was to investigate the difference between LES and NLES practices in achieving three QOF outcome measures:

1. DM12 (blood pressure, BP): Percentage of patients with diabetes in whom the last blood pressure was ≤ 145/85mmHg;
2. DM17 (lipid): Percentage of patients with diabetes whose last measured total cholesterol within the previous 15months was ≤5mmol/l;
3. DM23 (glycaemia): Percentage of patients with diabetes in whom the last HbA1c (DCCT aligned) was ≤7% (53 mmol/mol) (or equivalent test/reference range depending on local laboratory) in the previous 15months.

The secondary outcomes examined were:

1. Changes in achievement of indicators in 2004-2005 (prior to LES) compared to 2009-2010;
2. Hospital attendance for a first or follow-up diabetes related appointment for 2009–2010.

In 2004-2005, there was no DM23 (glycaemia) indicator. The equivalent was DM6: percentage of patients with diabetes in whom the last HbA1C was ≤7.4 % (57 mmol/mol) (or equivalent test/reference range depending on local laboratory) in last 15months. The targets set for HbA1c have been more stringent recently than in 2004-2005. DM6 is, however, the closest measure to DM23 (glycaemia) available. 

### Ethics Statement

This study, using practice performance data, was a service evaluation, and did not need any ethical approval as recommended by the UK National Research Ethics Service [17]. 

### Statistical Analyses

All analyses were carried out using the statistics package R. We used the odds ratio to test significant differences between LES and NLES practices. This is a measure of association of the group indicator (GP in LES vs. GP not in LES) with the occurrence of a given event (achieving the target QOF outcome) of 76 GPs first. The sampling units we used were the GP practices rather than individual patients. This helped to exclude the existence of patient confounding factors such as patient demographics, differences in the number of years doctors have been practicing or time of each consultation.

We cross-classified the total number of patients in the LES and NLES groups by patients who achieved (Positive) vs. the patients who did not achieve (Negative) the target. This allowed us to test whether LES practices achieved the targets more or less than NLES practices.

The group count data were reformatted to individual binary outcomes. We had adequate sample size of GPs for our investigations. We therefore did not use power to specify the sample size. 

A sample size of 21,026 patients in 76 GPs were included to investigate whether LES GPs performed better than NLES GPs in each of the QOF outcomes and for all outcomes combined. We used multilevel modeling to measure achievements for each patient nested within GP practices [18]. Combined analysis of performance in QOF outcomes was carried out to understand and evaluate QOF goals and achievements. We conducted logistic multilevel modeling on the combined structure of the three outcomes. The practice sizes of each GP are random as they vary between practices. GP practice size vanishes when formulating multilevel data structure.

We further explored improvement over time for 34 GPs. A sample of 34 NLES GPs were used to investigate (4,467 patients at the base time, and 6,253 at comparison) how each GP practice affects the probability of achieving the target over two time points in each of the three QOF outcomes, and all outcomes combined. We used Cochran-Mantel-Haenszel(CMH) 2X2Xk(k denotes GP, i.e. 34 GP)[19] Chi-Square to test and estimate the changes over two time points for DM12 (BP), DM17 (lipid) and DM23 (glycaemia). We then modeled the changes to the three odds ratio data over the two time point to investigate the change over time [19]. The GPs represent the group level and the patients stand for individual level data. The indicators for the three QOF indicator groups were used as a fixed effect factor. We further used a multilevel model to fit the three odds ratio data to answer the second part of the investigation of change over time. We started with the empty model “no fixed effect” first and then used the QOF indicator groups factor as fixed covariate.

For hospital attendance, we compared mean attendance of LES practices with NLES practices, for both a first and follow-up appointment. We used logistic regression to estimate the group effect (LES vs. NLES) on the probability of a new and follow-up hospital appointment. 

## Results

### The effect of LES on QOF indicators in 2009-2010

**Figure 1:**
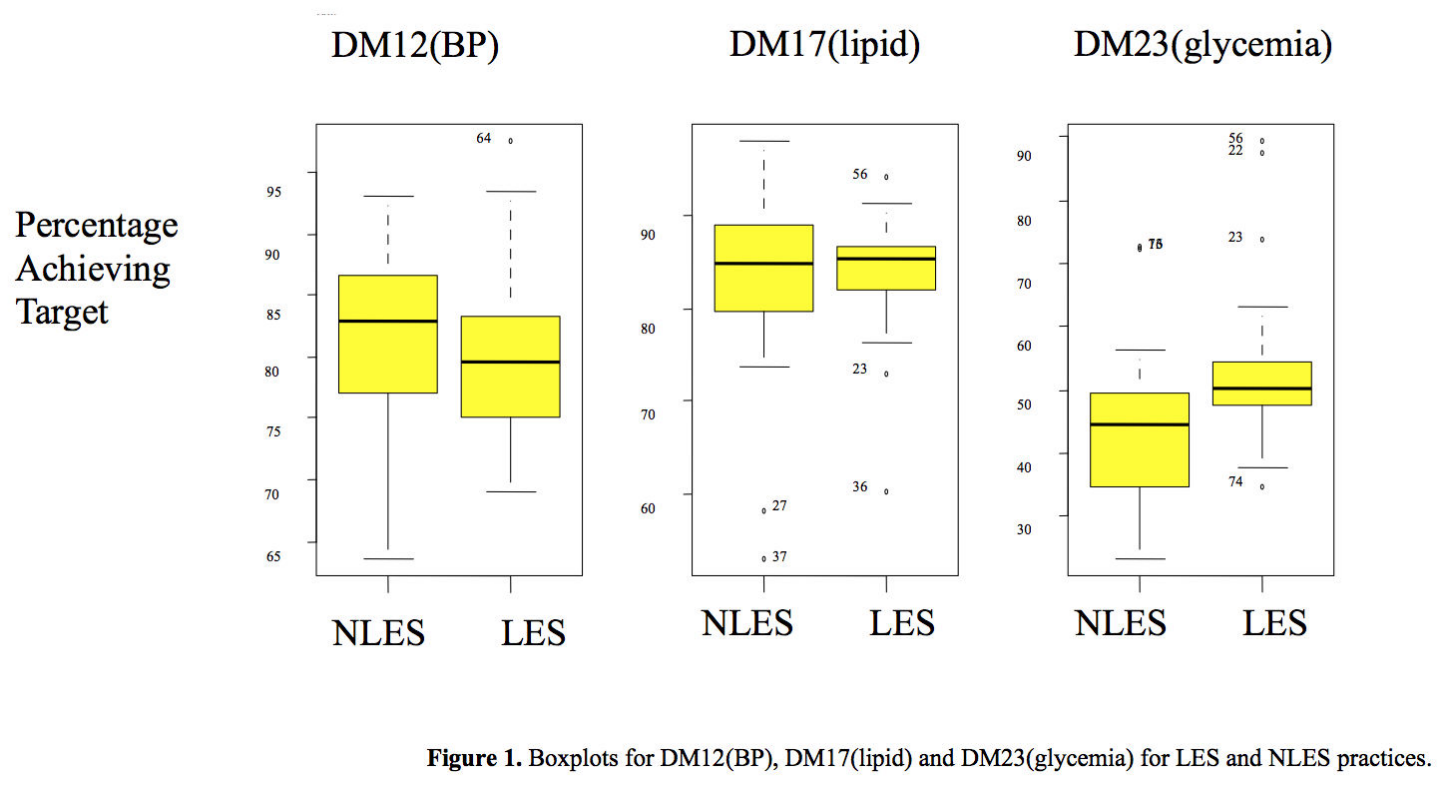
Boxplots for DM12(BP), DM17(lipid) and DM23(glycemia) for LES and NLES practices.

The percentage achieving the target in the 3 outcomes for the LES and NLES group are presented in Figure 1. Of all QOF targets, a lesser percentage of patients achieved the glycemic target, suggesting that glycaemia needs to be improved upon. DM12 (BP) shows higher achievement for the NLES group whereas DM17 (lipid) shows high percentage of achievements for both groups, although they did not differ significantly. The median in DM23 (glycaemia), however, was higher for the LES group practices than the NLES group. 

Table 1 presents results of LES and NLES groups cross-classified by achievement of targets. No statistically significant observations were obtained for DM12 (BP) and DM17 (lipid). The difference for DM23 (glycaemia) was, however, statistically significant (*P*=0.0001); LES practices were more likely to achieve the glycemic outcome. 

**Table 1:** The Odds ratio for the three outcomes, DM12 (BP), DM17 (Lipid) and DM23 (Glycaemia) (2x2 tables).

	**DM12 (BP)**		**DM17 (Lipid)**		**DM23 (Glycaemia)**	
	**Pos**	**Neg**	**Pos**	**Neg**	**Pos**	**Neg**
LES	10732	2671	11354	2049	7095	6308
NonLES	6174	1449	6404	1219	3319	4304
OR, p-value	0.943 (0.1097)		1.05 (0.1826)		1.459* (0.0001)	
CI	{0.878 1.013}		{0.976 1.140}		{1.378 1.544}	

**Figure 2:**
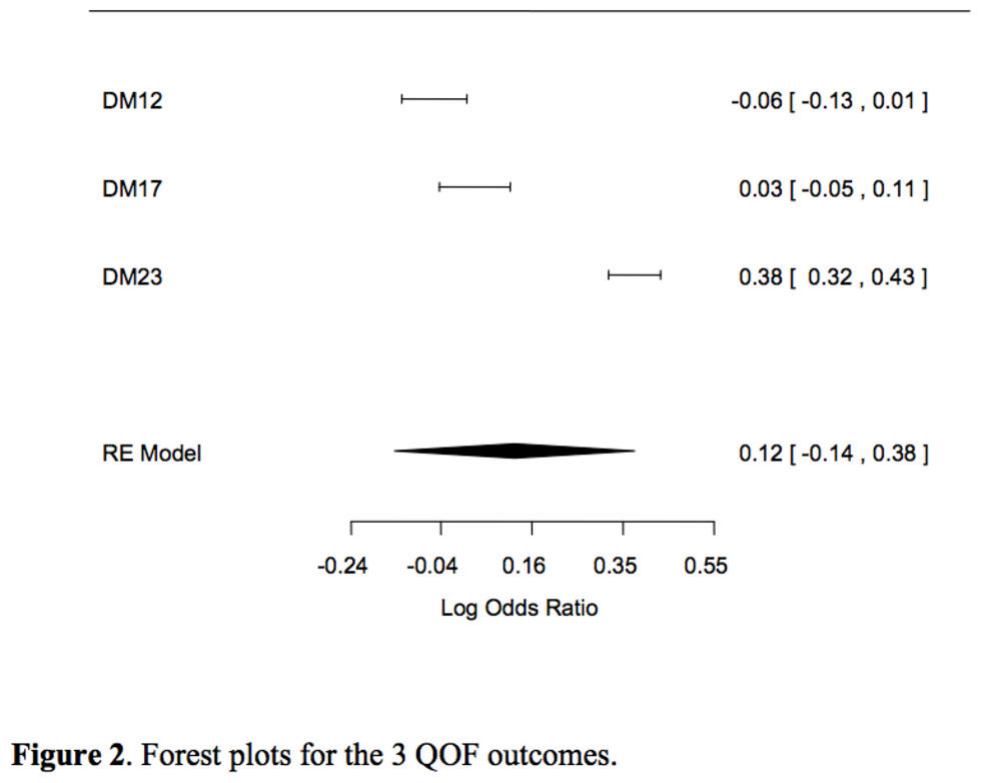
Forest plots for the 3 QOF outcomes.

The effect size (the term ‘effect size’ is used generically to denote the outcome measures DM12, DM17, DM23) (odds ratio; OR) for the 3 QOF outcomes was not independent. Therefore, the same sample was used to compute 3 ORs for LES vs. NLES practice achievements. The forest plots in Figure 2 describe the effect size of each QOF group and estimate the combination and its confidence interval. DM23 (glycaemia) effect size is positive and significant; the combination of the 3 outcomes is also positive but not significant. 

Table 2 presents results based on multilevel modeling approach in which the clustering effect of individual patients nested into GP practice were considered. The LES has significant effect on DM23 (glycaemia) measurement (hazard ratio 1.134;P<0.001) compared to NLES. The probability of achieving a satisfactory level of DM23 (glycaemia) increases by almost 10% for LES groups compared with the alternative. However, the effect of LES on DM12 (BP) and DM17 (lipid) was not significant.

**Table 2:** Results from fitting logistic multilevel modeling approach in which the clustering effect of individual patients nested into GP practice were considered.

**Parameter estimate**	**DM12 (BP)**	**DM17 (Lipid)**	**DM23 (Glycaemia)**
Random Effects Intercept	**0.197**	**0.164**	**0.215**
Fixed Effects Intercept	**1.561 (0.078)**	**1.690 (0.073)**	**-0.253 (0.078**)
Fixed Effects LES	**-0.117 (0.111**)	**0.027 (0.104)**	**0.385 (0.112)**
Loglikelihood	**-10197**	**-8979**	**-14144**

The LES has significant effect on DM23 (glycaemia) measurement (hazard ratio 1.134;P<0.001) compared to NLES. The effect of LES on DM12 (BP) and DM17 (lipid) was not significant.

The first part of Table 3 shows the general estimate of GP random effect and fixed effect of the intercept and LES (LES Model). Compared to individual groups in Table 2, the random intercept increased dramatically, indicating larger sample size and higher variance. The fixed intercept was significant and the LES effect not significant. In the second part (interaction model) we adjusted for the three QOF outcome groups and used the intercept at zero to avoid using the first item as the reference item and the basis for the intercept. There was no change in random intercept and the log likelihood when running the second part (interaction model). We had 6 significant estimates for the interaction of the QOF outcome group type with the LES indicator. The first three LES coefficients showed little difference, indicating that the probability of achieving the target is a function of the interaction of the LES group and the QOF indicator type. 

**Table 3:** The general estimate of GP random effect and fixed effect of the intercept and LES (LES Model; see text).

**LES Model**	**Estimate P-value**	**Interaction Model**	**Estimate P-value**
Random effects Intercept	1.9675		1.9674
Fixed effects Intercept	1.13573 (0.00001)		
Fixed effects LES	-0.03426 (0.91600)	DM12*LES	1.1372 (0.000001)
		DM17*LES	1.1343 (0.000001)
		DM23*LES	1.1358 (0.000001)
		DM12*NonLES	1.1015 (0.000001)
		DM17*NonLES	1.0988 (0.000001)
		DM23*NonLES	1.1054 (0.000001)
LogLik	-32967	LogLik	-32967

The LES has significant effect on DM23 (glycaemia) measurement (hazard ratio 1.134;P<0.001) compared to NLES. The effect of LES on DM12 (BP) and DM17 (lipid) was not significant.

The second three NLES significant interaction coefficients reflect similar results. The comparisons between LES and NLES interaction coefficient clearly show that LES GPs do better than NLES GPs with a very small margin.

### Testing changes over two time points

We compared available data from 34 of the NLES practices in 2004-2005 as a base compared to 2009-2010 as our comparison. The CMH test assumes that the odds ratios are near the same across GPs with asymptotic χ^2^ distributions. Thus we rejected the null hypothesis of conditional independence between the two time points given GP, see Table 4. The Q test for heterogeneity suggests considerable heterogeneity among the true performance. It is more informative to estimate the association than to test the hypothesis about it. 

**Table 4:** Q and CMH test statistic and its P-values (see text).

**Test statistic**	**DM12 (BP)**	**DM17 (Lipid)**	**DM23 (Glycaemia)**
Q test of heterogeneity (df)	120.49 (33)	95.28 (33)	136.15 (33)
P-value	<0.0001	<0.0001	<0.0001
CMH (df)	379.53 (1)	424.00 (1)	61.10 (1)
P-value	<0.0001	<0.0001	<0.0001

When modeling the changes over time, we identified a significant performance difference for DM12 (BP) between 2009-2010 and 2004-2005 (P<0.0001). The combined OR estimate was 2.27, indicating that achievement of DM12 (BP) in 2009-2010 is 2.27 times better than achievement in 2004-2005. For DM17 (lipid), we also found a significant difference between 2009-2010 and 2004-2005 (P<0.0001) and the combined OR estimate is 2.67 indicating that achievement of DM17 (lipid) in 2009-2010 is 2.67 times better than achievement in 2004-2005. With DM23 (glycaemia), there is a significant drawback in achieving the targets with a combined odds ratio estimate of 0.64. However, this may be because the targets for HbA1c are different now compared to 2004-2005. The targets have been set lower, and are more difficult to achieve. The results in Table 5 show the common OR estimate (12).

**Table 5:** Estimate and confidence intervals of common odds ratio, total amount of heterogeneity (τ^2^) and percentage of total variability due to heterogeneity (I^2^).

**Estimator**	**DM12 (BP)**	**DM17 (Lipid)**	**DM23 (Glycaemia)**
Common Odds ratio (C.I)	2.380 (1.962 - 2.890)	2.633 (2.223 - 3.121)	0.709 (0.591 - 0.849)
τ^2^ (C.I)	0.234 (0.120 - 0.486)	0.151(0.061 - 0.314)	0.215 (0.121 - 0.479)
I^2^ (C.I)	75.35 (60.94 - 86.36)	63.67 (41.54 - 78.49)	78.70 (67.56 - 89.16)

Table 6 shows the estimate of the multilevel model to fit the three OR data to answer the second part of the investigation of change over time. The difference in the loglikelihood of the empty and covariates model demonstrates improvement in the fitting with respect to the covariate model.

**Table 6:** Parameters estimate of two multilevel models.

**Parameter**	**Empty Model**	**Covariate model**
Random Effects- GP	0.0000	0.0926
Residual	0.6704	0.2178
Fixed Effects- Intercept	0.487 (0.081)	-0.363 (0.096)
DM12 (BP)		1.223 (0.113)
DM17 (Lipid)		1.325 (0.113)
Loglikelihood	-125.4	-83.89

The inter class correlation (ICC) of covariate model indicates that 30% of the variation is attributed to GP and 70% are attributing to individual patients. A significant difference between the two groups DM12 (BP) and DM23 (glycaemia) was recognized. The DM12 (BP) parameter estimate of (1.223) shows significant opposite direction. The DM17 (lipid) parameter estimate is even higher (1.325). 

### Diabetes related hospital attendance

**Figure 3:**
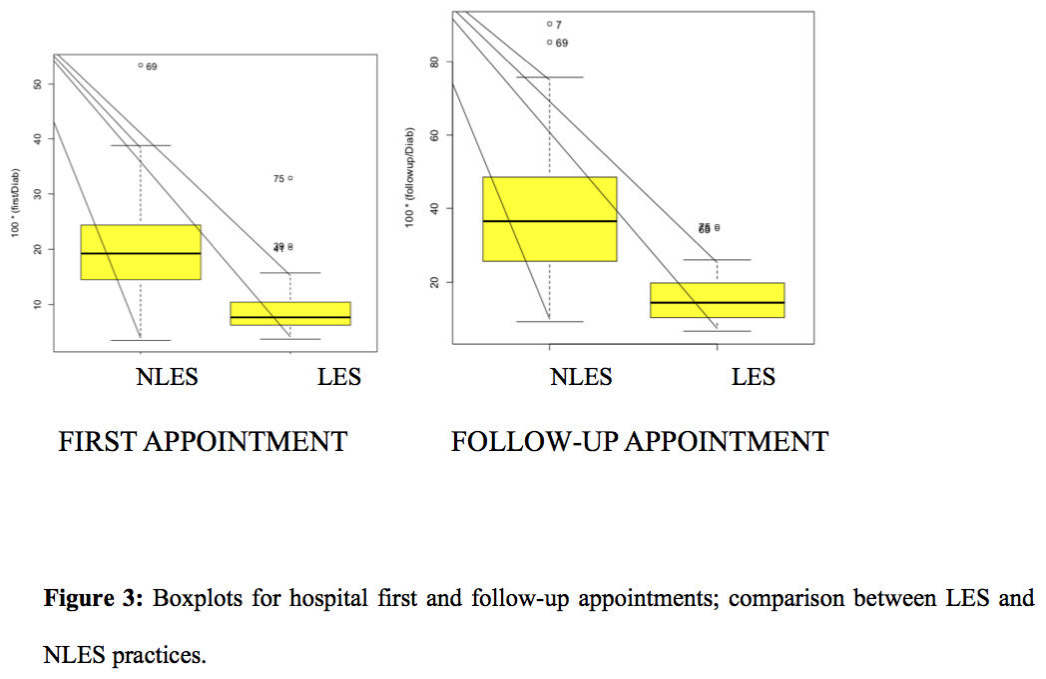
Boxplots for hospital first and follow-up appointments; comparison between LES and NLES practices.

For diabetes related hospital appointments, there was a significant difference between LES and NLES practices, with LES practices referring fewer patients for hospital appointments, both for a new appointment and also for follow-up appointments (Figure 3). The logistic regression estimate of the group effect (LES vs. NLES) on the probability of a new appointment is 0.69, i.e. OR is 1.21 higher for NLES compared to LES group. For the follow-up the probability estimate from logistic regression is 0.77, i.e. OR is 2.37 higher for NLES group compared to LES group. The above modeling takes the effect of one predictor at a time on the logit of the new appointment and follow-up. 

## Discussion

To our knowledge this is the first study to undertake a comparison of quality of care in locally enhanced diabetes programmes using QOF indicators. The establishment of local enhanced services was recommended to improve care for specific diseases. LES practices were expected to provide all essential and additional services that they were contracted to provide for patients with diabetes. The key finding of this study is that practices in the LES group were achieving significantly better targets for the QOF indicator DM23 (glycaemia) than NLES practices. However, there was no significant difference between DM12 (BP) and DM17 (lipid) indicators between LES and Non-LES practices. 

When we investigated the effects of time on achievement of target, it was difficult to conduct for DM23 (glycaemia).This was because in 2004-2005 there was no DM23 indicator. The closest QOF indicator was DM6, which had a higher HbA1c target. We identified that these practices were achieving better HbA1c outcomes in 2004-2005 than in 2009-2010. However, this may be due to the fact that HbA1c targets are more stringent in the latter time period, so more difficult to achieve than in 2004-2005. Over time, there was a significant improvement over time for DM12 (BP) and DM17 (lipid), indicating that general performance of practices has improved. Both of these results support previous research, which also found that between 1997 and 2005, the proportion of patients whose blood pressure and cholesterol met national targets increased in all sized practices [20]. 

The limitation of our study was that initial analyses considered only the patient variation and assumed that there was no variation among GPs, as if all the patients in the three QOF outcome measures belonged to one GP. The absence of an important variance component might preclude external generalizability of the results. Nesting data format can help in compensating for variance among GPs, i.e. patients nested within GPs. This new multilevel data structure provided good ground for running a different type of modeling. Multilevel models are used to make inference about the relationships between explanatory variables and response variables within and among GPs. This type of model simultaneously handles patient level relationships and takes account of the way patients are grouped in GPs. We found that the probability of achieving satisfactory level of DM23 (glycaemia) increases by almost 10% when GPs belong to LES groups compared with GPs in NLES groups, although the LES estimate of DM12 (BP) and DM17 (lipid) was not significant.

Longitudinal follow-up would have helped ascertain the effectiveness of LES further. However, longitudinal follow-up requires 3 time points to estimate the intercept and slope. Our study included 2 time points as this was the data available at the time of performing the analysis. 

For diabetes related hospital appointments, there was a significant reduction between LES and NLES practices, with LES practices referring fewer patients for hospital appointments. One reason for this may be that staff in LES practices are receiving more training and advice on better management of their diabetes patients, resulting in a reduction of complications and need for hospital referral. This is less likely, given the timeframe examined. The other reason is that in NLES practices, patients requiring insulin are referred to hospital for initiation and also follow-up. LES practices are required to manage patients on insulin, therefore reducing referral and follow-up appointments. This is also important in potentially explaining the greater achievement of glycemic targets by LES practices since glycemic control requires frequent and closer surveillance that can be provided more efficiently through local services.

In conclusion, our study shows that further financial incentivization and training through locally enhanced services can improve achievement of pay-for-performance glycemic targets [21], but not blood pressure and lipid targets. Given the increasing prevalence of diabetes and greater pressures on secondary care, exploring approaches such as LES should be examined further longitudinally. 
